# Synthesis of Medium-Sized Heterocycles by Transition-Metal-Catalyzed Intramolecular Cyclization

**DOI:** 10.3390/molecules25143147

**Published:** 2020-07-09

**Authors:** Mickael Choury, Alexandra Basilio Lopes, Gaëlle Blond, Mihaela Gulea

**Affiliations:** Université de Strasbourg, CNRS, Laboratoire d’Innovation Thérapeutique, LIT UMR 7200, F-67000 Strasbourg, France; mickael.choury@etu.unistra.fr (M.C.); alexandralopesb@yahoo.com.br (A.B.L.); gaelle.blond@unistra.fr (G.B.)

**Keywords:** transition-metal catalysis, intramolecular cyclization, medium-sized heterocycles

## Abstract

Medium-sized heterocycles (with 8 to 11 atoms) constitute important structural components of several biologically active natural compounds and represent promising scaffolds in medicinal chemistry. However, they are under-represented in the screening of chemical libraries as a consequence of being difficult to access. In particular, methods involving intramolecular bond formation are challenging due to unfavorable enthalpic and entropic factors, such as transannular interactions and conformational constraints. The present review focuses on the synthesis of medium-sized heterocycles by transition-metal-catalyzed intramolecular cyclization, which despite its drawbacks remains a straightforward and attractive synthesis strategy. The obtained heterocycles differ in their nature, number of heteroatoms, and ring size. The methods are classified according to the metal used (palladium, copper, gold, silver), then subdivided according to the type of bond formed, namely carbon–carbon or carbon–heteroatom.

## 1. Introduction

**Figure 1:**
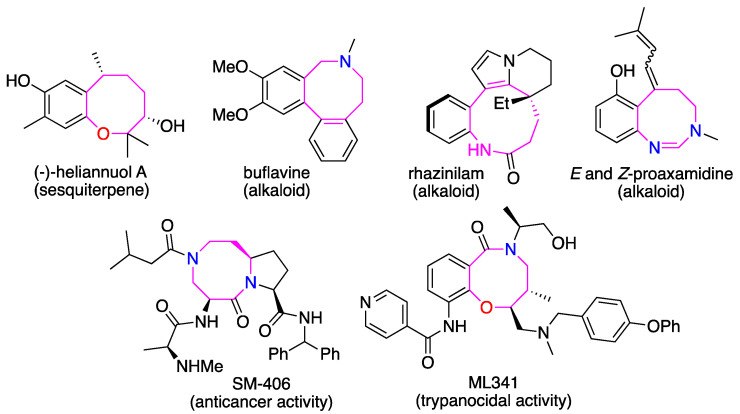
Examples of natural and bioactive compounds containing a medium-sized heterocycle.

Heterocyclic compounds are of major importance in pharmaceutical, agrochemical, and materials fields, and as synthetic tools. Among the large variety of methods to synthesize heterocycles, the transition-metal-catalyzed reactions have become a powerful, widely used strategy [1,2]. Most published articles relate to the use of transition metal catalysts for the synthesis of five-, six-, and even seven-membered heterocycles, while the medium-sized counterparts (i.e., eight- to eleven-membered heterocycles) [3,4] are sparsely reported. Indeed, the access to medium-sized rings is particularly difficult because of the high degree of transannular strain and unfavorable entropic factors involved [5,6], particularly when intramolecular cyclization strategies are used. Therefore, from a synthetic point of view, accessing medium-sized heterocycles still represents a challenge. Moreover, some of these heterocycles are structural components of diverse, biologically active natural products and pharmaceuticals (Figure 1).

Several excellent reviews have already covered the topic of synthesis of medium-sized rings, some of them dealing more specifically with ring-closing metathesis [7,8], radical-mediated reactions [9], annulations, ring expansion reactions [10,11,12], metal-mediated strategies [13,14,15,16,17], microwave-assisted synthesis [18]. Most of them were organized according to the type of the transformation involved to obtain the heterocycle and the structural elements of the heterocycle (i.e., ring size, type and number of heteroatoms).

The present review focuses on synthetic methods based on transition-metal-catalyzed intramolecular cyclization to access medium-sized heterocycles. The methods are classified according to the metal used as the catalyst (palladium, copper, gold, or silver), then subdivided according to the type of the formed bond, namely carbon–carbon or carbon–heteroatom (C–O, C–N, or C–S). The obtained heterocycles differ in their varied elements, such as the nature of the heteroatoms (O, N, S), the number of heteroatoms (one or more), and their ring size (8 to 11 atoms). The review mostly highlights recent literature (last decade), however some earlier publications are discussed when relevant to the topic or when they represent one of the rare examples described in a category.

## 2. Methods Using Palladium-Catalyzed Reactions

### 2.1. Carbon–Carbon Bond Formation

#### 2.1.1. Intramolecular Heck Reaction

**Scheme 1:**
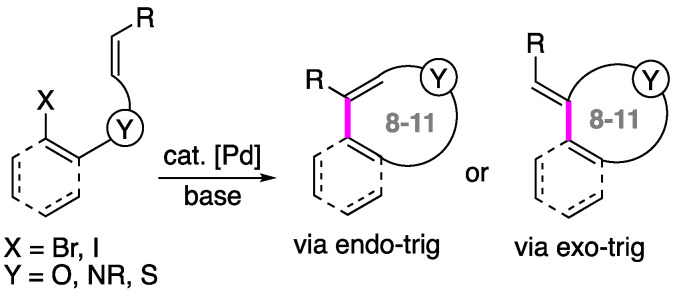
Schematic representation of an intramolecular Heck reaction accessing medium-sized heterocycles.

The intramolecular variant of the Heck reaction consists of the palladium-catalyzed coupling of an aryl or alkenyl halide with an alkene in the same molecule, leading to a carbocyclic or a heterocyclic structure bearing an *endo* or *exo* double bond resulting from β-hydride elimination in the final step. In the category of cross-coupling reactions, this method is probably the most encountered for accessing medium rings. Among the various substrates designed for this reaction, some have been appropriately tethered to afford medium-sized heterocycles (Scheme 1). Generally, in these cases *endo*-trig cyclization is favored, but depending on the substrate structure competing *endo* or *exo* cyclization could be encountered.

The literature dealing with the synthesis of medium-sized heterocycles by intramolecular Heck cyclization has been covered until 2011, mostly by two reviews published by Majumdar [16,17]. To avoid redundancy, we decided to not discuss the publications having been reported therein in the present review. However, to show the wide diversity of structures obtained by this strategy, selected examples of obtained products (i.e., 8 to 10 membered-sized *O*-, *S*-, *O, S*- or *N*-heterocycles) have been summarized in Table 1, with the corresponding data (reaction conditions, yields, number of examples) extracted from the cited articles. Two more recent examples that were not covered by the reviews mentioned before were also added (Table 1, entries 7 and 14).

#### 2.1.2. Intramolecular Pd-Catalyzed Cyclization of Alkynes

The most encountered reaction in this category is the intramolecular carbopalladation of alkynes, which represents a version of the Heck–Mizoroki reaction using an alkyne instead of an alkene [32,33,34]. A vinyl–palladium species is generated in this catalytic process and can be trapped by reduction or cross-coupling. In our context, this generally involves an acyclic substrate bearing aryl, vinyl iodide, or bromide, and a triple bond tethered by an appropriate chain, including at least one heteroatom. Placed under Pd catalysis conditions, this type of substrate can afford a medium-sized heterocycle, which includes an *exo* or *endo* double bond with stereo-controlled geometry. A competing direct reduction (or C–C cross-coupling) might be observed, given the difficulty of alkyne intramolecular carbopalladation to form medium-sized rings.

**Scheme 2:**
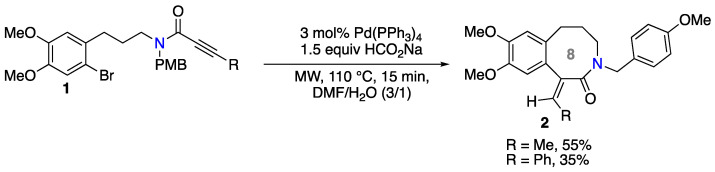
Synthesis of benzo[*d*]azocines by reductive cyclocarbopalladation of alkynes.

Van der Eycken and Donets reported a regio- and stereoselective reductive cyclocarbopalladation of propargylamides to synthesize 3-benzazepines, which are 7-membered *N*-heterocycles [35]. Then, the authors demonstrated that the method could be extended to the synthesis of the 8-membered ring counterparts, namely benzoazocines. Substrate **1** was placed under microwave-assisted conditions at 110 °C, in a mixture of dimethylformamide-water, in the presence of palladium-tetrakis(triphenylphosphine) as the catalyst (Scheme 2). Sodium formate was used as the H-donor for the reduction. The 8-*exo*-dig cyclization via *syn*-addition of the arylpalladium species to the triple bond exclusively provided the desired product **2** in moderate yields, with Z-stereochemistry of the exocyclic double bond.

**Scheme 3:**
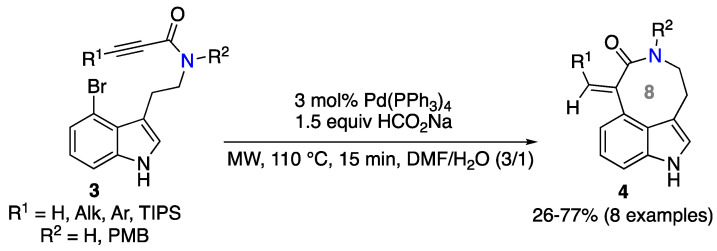
Synthesis of azocino[*cd*]indoles by reductive cyclocarbopalladation of alkynes.

The same research group applied this methodology to access azocino-[*cd*]indoles [36]. Starting from substrates **3**, the reaction was performed under Pd catalysis conditions, using microwave heating at 110 °C, in DMF–water, and led with complete conversion after 15 min to the desired product **4** in good yields (Scheme 3). As in the previous cases, the reaction occurred with total regioselectivity in favor of the 8-*exo*-dig cyclization and with full control of the geometry around the exocyclic double bond.

**Scheme 4:**
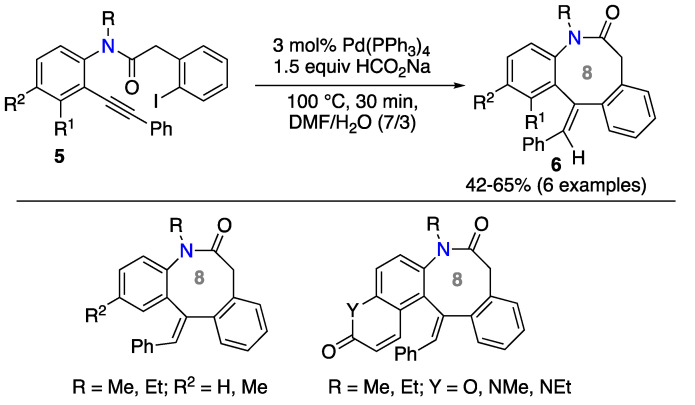
Synthesis of dibenzoazocines by reductive cyclocarbopalladation of alkynes.

Another application of the method was reported by Majumdar and co-workers, who synthesized dibenzoazocine derivatives [37]. The reaction conditions were similar to those previously reported but performed under conventional heating. The alkynyl group is directly linked to the aromatic ring of substrate **5** and the *ortho*-iodobenzene moiety is placed as a substituent on the amide chain. From this, diverse dibenzoazocine **6** products were obtained in satisfactory yields (Scheme 4).

**Scheme 5:**
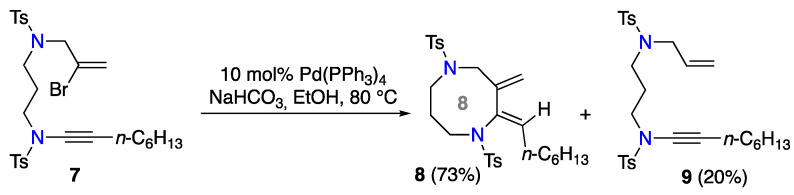
Pd-catalyzed reductive cyclization of bromoenynamides.

Anderson and co-workers reported the reductive Pd-catalyzed cyclization of bromoenynamides and used ethanol as the hydride source [38]. The transformation afforded 2-amido exocyclic dienes with a ring size of 5 to 8. The example leading to the 8-membered ring product diazocane 8 is depicted in Scheme 5. The competing direct reduction of **7** was observed in this case, however, the corresponding product **9** was minor in the mixture with the desired heterocycle (the ratio **8/9** was 8:2).

**Scheme 6:**
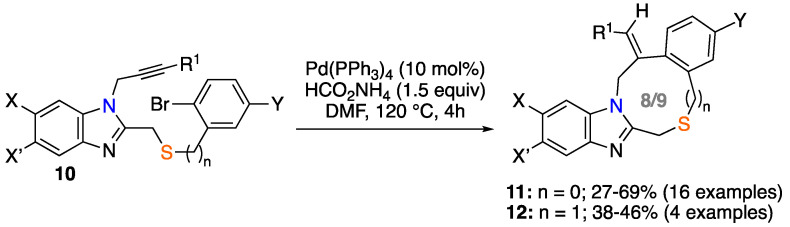
Pd-catalyzed reductive cyclization to access thiazocine and thiazonine derivatives.

The synthesis of sulfur-containing heterocycles via palladium-catalyzed reactions is a less-common method than for the other heterocycles, because sulfur species are known to deactivate the Pd catalyst and make the reaction difficult. Our group was interested in this aspect, and since 2014 has published several papers dealing with the use of intramolecular carbopalladation of propargyl or alkynyl sulfides to access 5- and 6-membered *S*-heterocycles [39,40,41]. Then, we decided to extend the method to more challenging compounds, such as the medium-sized rings, and focused on *N*,*S*-heterocycles as target compounds. We first developed the synthesis of benzimidazole-fused thiazocine **11** and thiazonine **12** via the 8- or 9-*exo*-dig reductive Pd-catalyzed cyclization, respectively, starting from 2-sulfanylated benzimidazole derivative **10** (Scheme 6) [42]. Ammonium or sodium formate was used as the reducing agent. The direct reduction of **10** was a competitive reaction, however the resulting side product was minor and could be removed by column chromatography and the desired cyclic products isolated in satisfactory yields. By varying the substituents on the substrate; R^1^ (alkyl, aryl, heteroaryl) on the triple bond; or X, X’, Y on the aromatics, the scope of the reaction was demonstrated to be broad.

**Scheme 7:**
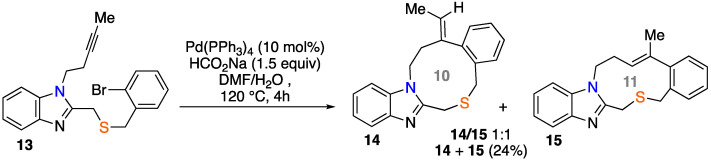
Pd-catalyzed reductive cyclization to access thiazecine and thiazaundecine derivatives.

During the same study, when we attempted to extend the method to access 10-membered rings, the major product obtained was the one resulting from the reduction of substrate **13** (isolated in 49% yield). However, the crude mixture also contained two inseparable cyclic products, thiazecine **14** and of thiazaundecine **15**, at a ratio **14/15** of 1:1 and with a combined yield of 24% (Scheme 7). This represents a rare example of competing processes between 10-*exo* and 11-*endo* cyclocarbopalladation of alkynes.

We also attempted to trap the vinyl-palladium intermediate by a Suzuki–Miyaura coupling instead of the reduction to produce benzimidazole-fused thiazocines bearing a stereo-defined tetrasubstituted exocyclic double bond; however, the yield was very low [43].

**Scheme 8:**
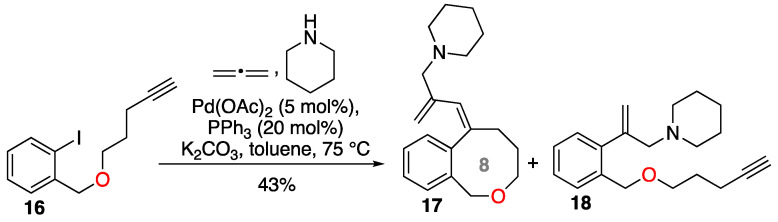
Pd-catalyzed cyclization–allenylation–amination to access tetrahydro-2-benzoxocine.

A nice transformation based on the intramolecular *exo*-dig cyclization strategy was described in 2002 by Grigg and co-workers [44]. The cyclocarbopalladation is followed by an allene insertion and final capture of the resulting π-allyl palladium(II) species by a secondary amine nucleophile. The authors explored the possibility of forming an eight-membered ring using this methodology; therefore, one example was achieved starting from substrate **16**, affording the desired tetrahydro-2-benzoxocine **17** and the acyclic product **18** resulting from the direct coupling in a 1:1.4 mixture, with a 43% combined yield (Scheme 8).

**Scheme 9:**
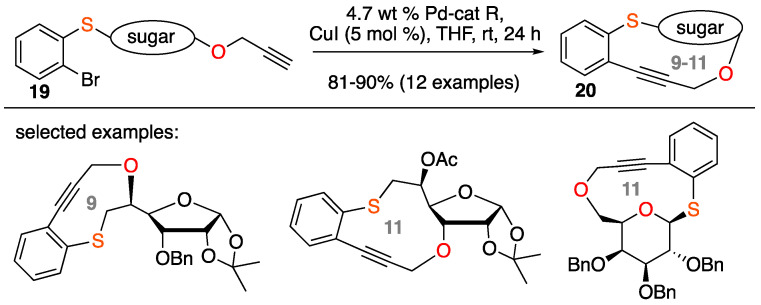
Intramolecular Sonogashira reaction to access medium-sized *O,S*-heterocycles.

In 2013, Mukherjee and co-workers described intramolecular Sonogashira cross-coupling using judiciously substituted sugars to access medium-sized *O,S*-heterocycles [25]. Various sugar-based *O*-propargyl derivatives were prepared, including propargyl ethers derived from 1,2,5,6-di-*O*-acetonide-α-D-glucofuranose and 1,2,3,4-di-*O*-acetonide-α-D-galactopyranoside (Scheme 9). The reaction was performed with a heterogeneous palladium catalyst and copper iodide as the co-catalyst. Heterocyclic products with nine to eleven atoms and containing an endocyclic triple bond were obtained with good yields.

### 2.2. Carbon–Heteroatom Bond Formation

#### 2.2.1. C–N Bond Formation

Palladium-catalysis is extensively used in C–N bond formation [45,46]. Many reactions belonging to this category are applicable in an intramolecular version, leading to various N-heterocycles. In the examples highlighted hereafter, an intramolecular Pd-catalyzed amination was used to access medium-sized *N*-heterocycles.

Due to the advances in the field made independently by Buchwald and Hartwig, the Pd-catalyzed *N*-arylation is used for the synthesis of a wide range of acyclic or cyclic nitrogen-containing compounds; however, few examples are described for obtaining medium-sized *N*-heterocycles.

**Scheme 10:**
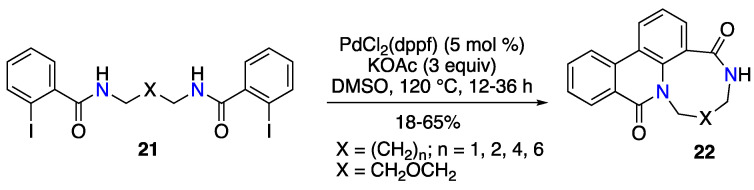
Synthesis of medium-sized *N-*polyheterocycles by a Pd-catalyzed intramolecular *N*-arylation–C–H activation–aryl–aryl bond-forming domino reaction.

An unprecedented intramolecular Buchwald–Hartwig amidation enabling the formation of medium-sized *N*-polyheterocycles was described by Zhu and co-workers [47]. The method consisted of a Pd-catalyzed domino reaction involving an intramolecular *N*-arylation, a C–H activation, and an aryl-aryl bond-formation, which led to *N*-polyheterocycle **22** with 8–11 and 13-membered rings (Scheme 10). The optimal catalytic reaction conditions were PdCl_2_(dppf) and KOAc in DMSO, but the authors also showed that starting from the substrate **21** with the ether tether, the ligand-free transformation using only Pd(OAc)_2_ was possible, probably due to an internal Pd coordination in a catalytic intermediate.

**Scheme 11:**
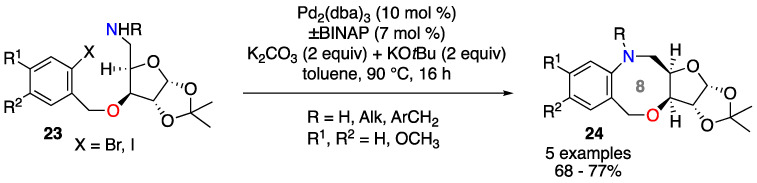
Synthesis of benzoxazocines by intramolecular Pd-catalyzed *N*-arylation.

Chattopadhyay and co-workers reported the synthesis of sugar-fused 8-membered *N*,*O*-heterocycles (Scheme 11) [48]. Starting from D-glucose, the suitable substrates **23** were functionalized with both an aryl halide and a secondary amine. Using classic conditions for the palladium-catalyzed *N*-arylation with Pd_2_(dba)_3_ as the precatalyst source and racemic (2,2′-bis(diphenylphosphino)-1,1′-binaphthyl) (BINAP) as the ligand, the substrates were converted with good yields into the corresponding benzoxazocine **24**.

**Scheme 12:**
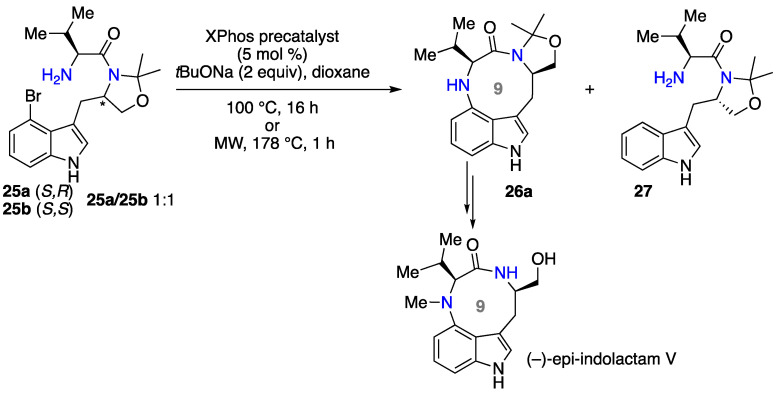
Synthesis of 9-membered *N*-heterocycle by intramolecular Pd-catalyzed *N*-arylation.

Piersanti and co-workers also used intramolecular N-arylation for the synthesis of a nine-membered *N*-heterocycle, the natural product (–)-epi-indolactam V. Starting from the appropriate precursor **25** derived from tryptophan, it was difficult for the reaction to succeed, and after many tested reaction conditions the best result was obtained using a palladacycle precatalyst based on XPhos phosphine ligand, NaOtBu as the base, and dioxane as the solvent (Scheme 12) [49]. The reaction could be accelerated using microwaves and by increasing the temperature. The initial experiment on the diastereomeric 1:1 mixture of precursors **25a** and **b** showed that the intramolecular *N*-arylation using these conditions was highly stereospecific, revealing the importance of a favored conformational preorganization induced by the stereocenters on the efficiency of the cyclization. Indeed, diastereoisomer **25a** was transformed into the desired heterocycle **26a**, while diastereoisomer **25b** furnished only traces of the corresponding heterocycle **26b**, along with the side product **27** resulting from reductive dehalogenation. Then, under the same conditions, the bromotryptophan dipeptide derivative **25a** was converted into the desired product **26a**, which was transformed in two steps into the natural product (-)-epi-indolactam V, with 81% overall yield.

**Scheme 13:**
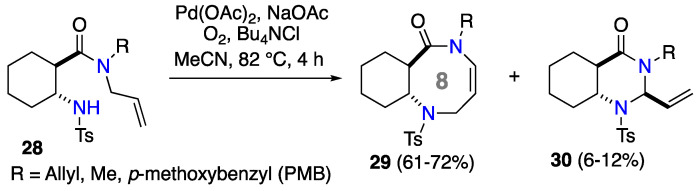
Synthesis of 8-membered *N*,*N*-heterocycles by Pd-catalyzed oxidative *cis*-aminopalladation.

A very well-documented study in the synthesis of cyclohexane-fused 1,5-diazocin-6-one was reported by Fülöp and co-workers using a Pd(II)-catalyzed oxidative intramolecular *cis*-aminopalladation reaction [50]. Starting from tosyl-protected *trans*-*N*-allyl-2-aminocyclohexanecarboxamides **28**, the optimization of the reaction conditions allowed a highly regioselective formation of the medium-sized heterocycles **29** via an 8-*endo*-trig cyclization, with relatively good yields (Scheme 13). Interestingly, this regioselectivity was enhanced by the solvent, allowing reduction of the amount of cyclohexane-fused pyrimidin-4-one product 30. On the other hand, this regioselectivity was observed only when the *trans* substrate was used, whereas the *cis*-isomer gave only the six-membered ring product.

**Scheme 14:**
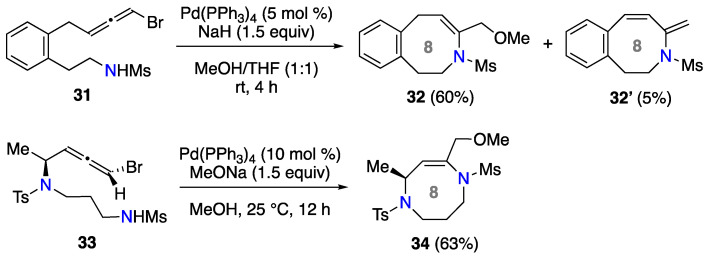
Synthesis of 8-membered *N*-heterocycles by Pd-catalyzed cyclizations of bromoallenes.

An interesting method to synthesize eight-membered *N*-heterocycles was reported by Ohno and co-workers, using as cyclization precursors bromoallene bearing a chain functionalized with a nitrogen nucleophile (Scheme 14) [51]. First, achiral bromoallene **31** attached to a sulfonamide group was prepared and converted under Pd catalysis in the presence of methanol into benzazocine **32** in good yield. A small amount of the side product **32′** was formed, corresponding to the β-elimination product. Then, applied to an enantiopure substrate **33**, the reaction took place regio- and stereo-selectively, leading to the optically active diazocine **34** in 63% yield.

**Scheme 15:**
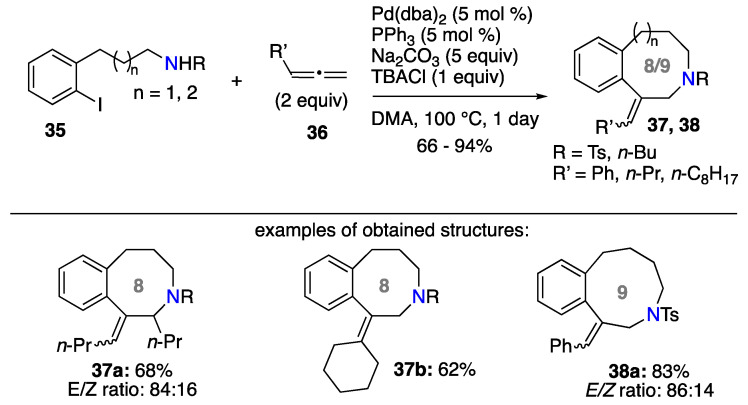
Synthesis of 8- or 9-membered *N*-heterocycles by Pd-catalyzed heteroannulation of 1,2-dienes.

In 1998, Larock’s group reported the synthesis of azepines containing an exocyclic double-bond by heteroannulation of allenes starting from amines or tosylamide derivative **35** attached to an aryl iodide moiety (Scheme 15) [52]. The Pd catalysis conditions allowed the insertion of the *mono*- or *di*-substituted allene **36** after the oxidative addition, generating a π-allylpalladium species. Then, a regioselective intramolecular attack at the non-substituted carbon of the π-allyl system by the *N*-nucleophile furnished the eight- or nine-membered ring. Products **37** or **38** were obtained in good yields as a mixture of *E/Z* isomers, with the *E*-isomer being the major one.

**Scheme 16:**
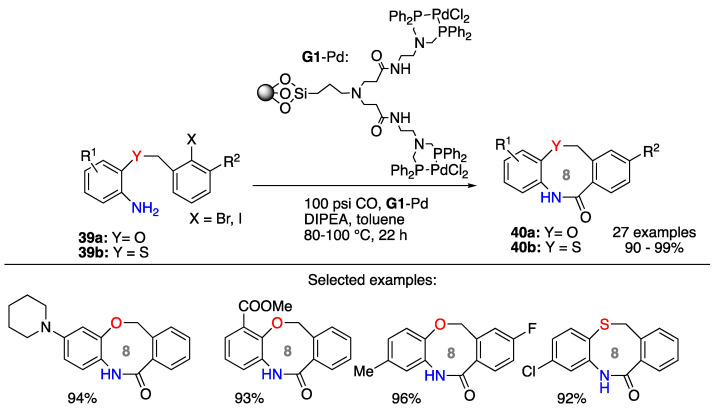
Synthesis of 8-membered *N*-heterocycles by Pd-catalyzed intramolecular carbonylation.

Another strategy was employed by Alper and Lu, who used palladium-complexed dendrimers supported on silica as catalysts to obtained 8-membered *N*,*O* and *N,S*-heterocycles by intramolecular carbonylation of aniline derivatives (Scheme 16) [53]. This recyclable source of palladium (**G1**-Pd) allowed in a first step the insertion of the carbon monoxide on various **39a** 2-((2-halobenzyl)oxy)anilines or the thioether analogue **39b**. Then, the intramolecular attack of the aniline nitrogen on the allylpalladium intermediate and C–N bond formation by reductive elimination furnished the desired 8-membered *N*,*O* or *N,S*-heterocycle **40** with excellent yields. This methodology allowed either strongly electron-withdrawing or electron-donating substituents on the anilines.

#### 2.2.2. C–O Bond Formation

**Scheme 17:**
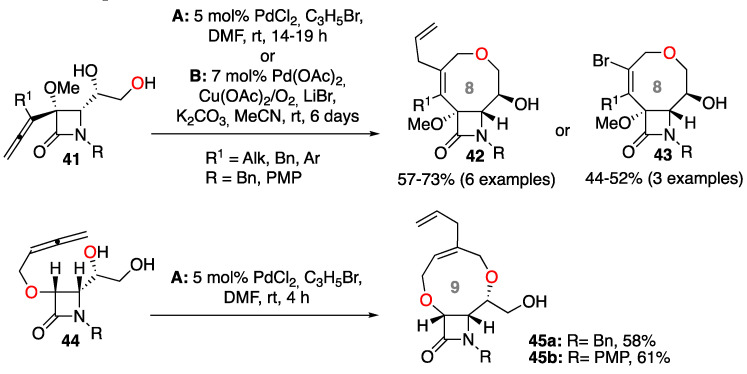
Synthesis of medium-sized *O*-heterocycles by Pd-catalyzed cyclization of allendiols.

Alcaide and co-workers developed a Pd-catalyzed intramolecular C–O bond formation by reacting 2-azetidinone-tethered allendiols with allyl bromide or lithium bromide, leading to azetidinone-fused 8- and 9-membered *O*-heterocycles [54]. Starting from enantiopure γ,δ-allendiols **41**, the 8-*endo* cyclization took place with total chemo- and regioselectivity via the attack of the primary hydroxy group to the terminal allene carbon, leading to oxocine **42** or **43**, depending on the reaction conditions **A** or **B** (Scheme 17). When ε,ζ-allendiols **44** were used as substrates, 2-azetidinone-fused dioxonines **45** were obtained via the 9-*endo* cyclization, under conditions A. Plausible mechanistic hypotheses were given by the authors and DFT studies have been performed to understand the experimental results.

#### 2.2.3. C–S Bond Formation

**Scheme 18:**
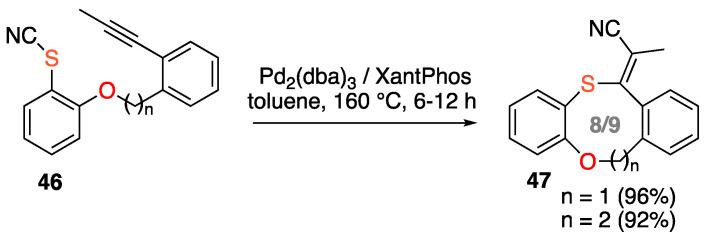
Synthesis of medium-sized *O,S*-heterocycles by Pd-catalyzed cyanosulfenylation.

Very recently, Werz and co-workers described the first example of Pd-catalyzed intramolecular cyanosulfenylation of a triple bond and applied this method to the synthesis of two medium-sized heterocycles **47**, one oxathiocin and one oxathionin (Scheme 18) [55]. Mechanistically, the sequence consists of a thiopalladation–cyanide transfer cascade.

### 2.3. Cyclization of Bromoallenes

**Scheme 19:**
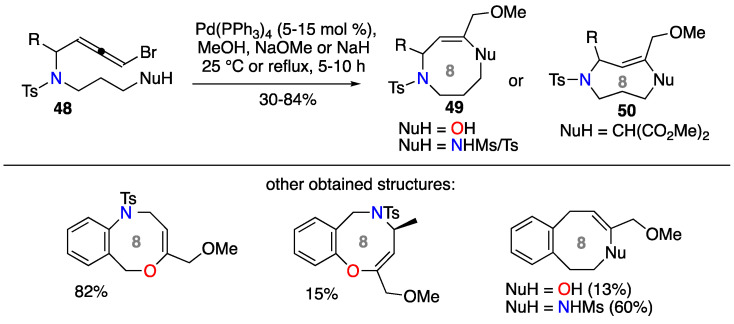
Synthesis of 8-membered heterocycles by Pd-catalyzed cyclization of bromoallenes.

Ohno, Tanaka, and co-workers described an original synthetic method to construct medium-sized rings consisting of the Pd-catalyzed cyclization of bromoallenes judiciously attached to a nucleophile, such as **48** [51]. In this section, we treated this case separately, as the three types of intramolecular bond formations are described (i.e., C–C, C–N, and C–O) by using a carbon, nitrogen, or an oxygen nucleophile, respectively, to attack the allene moiety (Scheme 19). The reaction took place in the presence of a Pd(0) catalyst and in methanol as the solvent. In this process, bromoallenes act as allyl dication equivalents and the intramolecular nucleophilic attack takes place exclusively at the central carbon. Interestingly, bromoallene **48** has a *N*- or an *O*-nucleophile with eight-membered rings, in which the double bond is of *cis*-geometry (product **49**), while those having a *C-*nucleophile give the corresponding *trans*-rings (products **50**).

### 2.4. Formal Cycloaddition

In the context of this review, the so-called “formal cycloadditions” do not belong strictly speaking to the category of metal-catalyzed intramolecular cyclization reactions, however, many of them fit with this definition. The selected examples of formal cycloaddition [n + m] are Pd-catalyzed processes occurring via the formation in a first step of an acyclic palladium-intermediate, followed by an intramolecular carbon-heteroatom bond formation leading to a medium-sized heterocycle with (n + m) atoms.

**Scheme 20:**
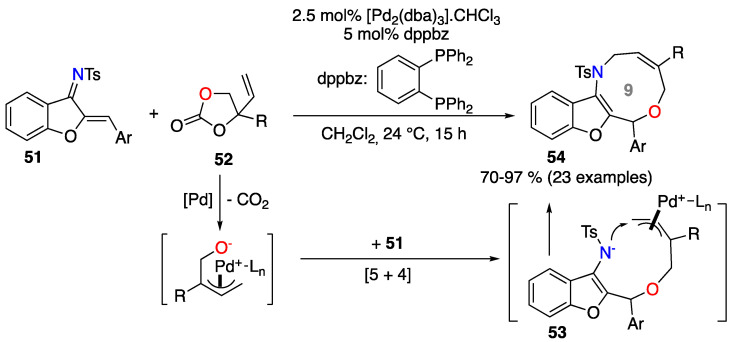
Synthesis of 9-membered *N*,*O*-heterocycles by Pd-catalyzed [5 + 4] formal cycloaddition.

Zhao and co-workers reported an elegant strategy to access nine-membered *N*,*O*-heterocycles via a formal [5 + 4] cycloaddition [56]. Azadiene **51** derived from benzofuran as the four-atom unit and the substituted vinylethylene carbonate (VEC) **52**, which is the palladium π-allyl alcoholate five-atom unit precursor, react under palladium-catalysis to generate species **53**. The reaction proceeds via the attack of the nitrogen-nucleophile to the terminal carbon of the Pd-π-allyl moiety to deliver product **54** (Scheme 20). The reaction scope proved to be broad with respect to the variation of substituents of both azadiene and VEC, leading to a wide range of benzofuran-fused oxazonines.

The same group developed then a catalytic enantioselective version of the reaction by using Pd catalysts with chiral diphosphine ligands [57]. The benzofuran-fused nine-membered heterocycles were obtained in excellent yield (70–95%) and with high enantioselectivity (86−92% ee).

**Scheme 21:**
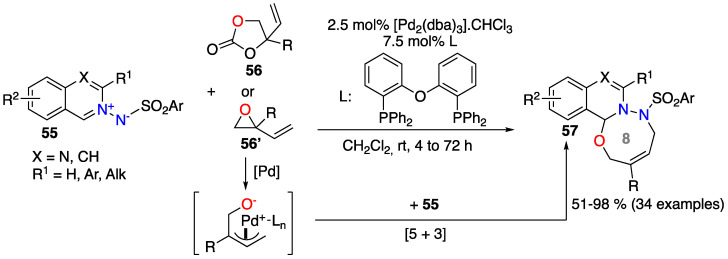
Synthesis of 8-membered *N*,*O*-heterocycles by Pd-catalyzed [5 + 3] formal cycloaddition.

According to a new strategy that used a 1,3-dipole as a three-atom partner, a formal [5 + 3] cycloaddition was developed by Guo’s group [58]. The zwitterionic allylpalladium intermediates were generated either from vinylethylene carbonates **56** or vinyloxiranes **56’** and reacted in situ with azomethine imines **55** to afford eight-membered *N*,*O*-heterocycle **57** in good to excellent yields (Scheme 21). *N*-quinazolinium and *N*-isoquinolinium ylides were successfully employed in the reaction, while other azomethine imines were inert. It should be noted that the regioselectivity of the reaction, namely [5 + 3] vs. [3 + 3] cycloaddition, was not complete; however, the formation of the 8-membered ring products was clearly favored (90:10 to 99:1 ratios). For non-substituted vinylethylene carbonate and vinyloxirane (with R = H), the 6-membered ring cycloadduct was obtained as the major product.

**Scheme 22:**
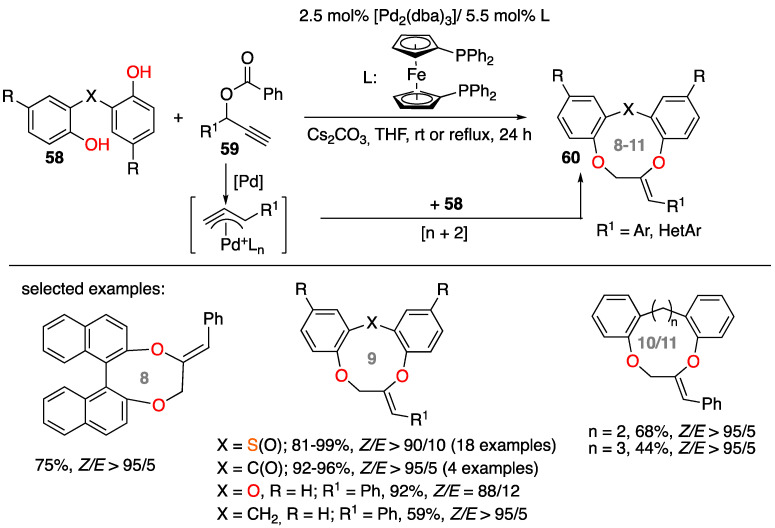
Synthesis of 8- to 11-membered heterocycles by Pd-catalyzed [n + 2] formal cycloaddition.

Another strategy that was recently published by Liu and Hu consisted of a Pd-catalyzed [n + 2] formal cycloaddition enabling access to medium-sized heterocycles from eight to eleven-membered rings [59]. Various linker-tethered-bisphenols **58** were used as n-atom (n = 6 to 9) bis-nucleophile partners in reaction with propargylic esters **59** as C2 synthons. Under palladium catalysis conditions, the propargylic benzoate affords a η3-π-propargylpalladium complex, which reacts with the bis-*O*-nucleophile to form the cyclic product **60** (Scheme 22). The reaction shows a broad substrate scope (particularly in 9-membered ring series), excellent regio- and *Z/E*-selectivities (>95/5), and high yields.

## 3. Methods Using Copper-Catalyzed Reactions

Copper-mediated and copper-catalyzed coupling reactions were pioneered by Ullmann and Goldberg at the beginning of the 20th century [60]. In its “classical” version, a C–C bond is formed via reductive coupling of haloarenes to give biaryl compounds. This principle was then extended to other nucleophiles, and now various heteroatom-aryl (heteroatom: N, O, S, P) and carbon-aryl bonds are easily accessible via this process. In particular, these reactions have been improved via the use of copper ligands, allowing lower catalyst loading and temperatures and broader substrate scope [46,61,62,63]. Copper oxidation states can range from Cu(0) to Cu(III). The use of copper catalysts in the synthesis of medium-sized heterocycles, although rare, has many advantages, since copper is abundant, inexpensive, air-stable, and compatible with different functional groups.

### 3.1. Carbon–Carbon Bond Formation

**Scheme 23:**
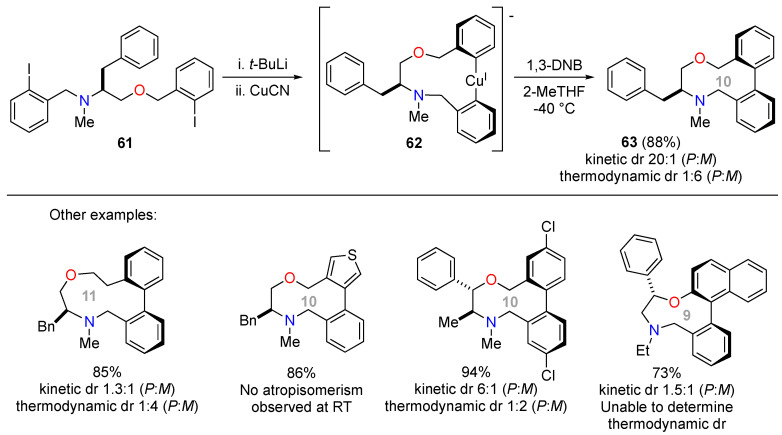
Stereoselective synthesis of biaryl-containing medium-sized heterocycles via an intramolecular copper-mediated C–C bond formation.

Schreiber and co-workers explored a branching reaction pathway strategy for a diversity-oriented synthesis (DOS) approach to build biaryl-embedded 9-, 10-, and 11-membered heterocycles via an intramolecular copper-catalyzed C–C bond formation [64]. As an example, the starting chiral aminoether **61** was treated with *t*-BuLi followed by CuCN to give the supposed cyclic organocuprate intermediate **62**, which under oxidizing conditions afforded biaryl atropisomeric 10-membered *N*,*O*-heterocycle **63** at 88% yield (Scheme 23). It has been shown that the nature of the substrate and the reaction conditions (oxidizing agent, solvent, and temperature) influenced the diastereoselectivity. Under optimized conditions, using 1,3-dinitrobenzene (1,3-DNB) as the oxidant and 2-MeTHF as the solvent at −40 °C, good yields were obtained in all cases. Thermal isomerization was used to reverse the stereochemistry of the major atropoisomer obtained in kinetic conditions. The diastereomeric ratios were measured for the products obtained under kinetic conditions (kinetic dr), then after heating each product at 150 °C for 24–48 h (thermodynamic dr). Considering a future split-pool synthesis, the authors judiciously extended the concept to a solid-phase process.

**Scheme 24:**
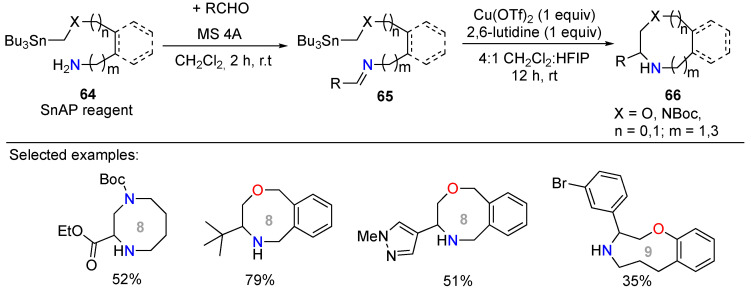
Synthesis of 8- to 9-membered heterocycles by Cu-catalyzed cyclization involving Sn amino protocol (SnAP) reagents.

Bode’s group developed what they called the Sn amino protocol (SnAP) reagents and employed them in the synthesis of functionalized saturated heterocycles [65]. The transformation occurred under mild reaction conditions and consisted of the condensation of an aldehyde and the SnAP reagent **64**, affording the imine intermediate **65**, which was then engaged in an oxidative radical process catalyzed by copper. Cyclization occurred via C–C bond formation through an *endo* attack to the imine bond by the stabilized radical cation formed in the α-position of heteroatom X. The catalytic cycle ends with the reduction of the cyclic *N*-radical cation by Cu(I) and regeneration of the Cu(II) catalyst. This approach was compatible with aliphatic, aryl, and heteroaryl aldehydes, allowing access to various 8- and 9-membered saturated *N*,*N*- and *N*,*O*-heterocycle **66** products (Scheme 24).

**Scheme 25:**
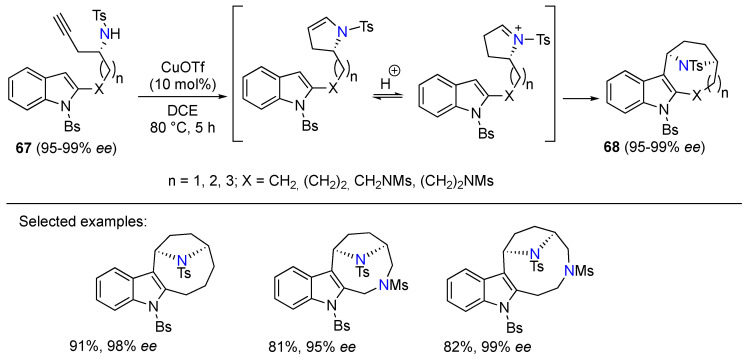
Synthesis of bridged aza-[*n*.2.1]-indolyl medium-sized heterocycles by copper-catalyzed hydroamination–Friedel–Crafts domino process.

Ye and co-workers disclosed the first copper-catalyzed tandem reaction of chiral indolyl homopropargyl amide **67** involving a 5-*endo-*dig hydroamination and a subsequent Friedel–Crafts alkylation [66]. This method afforded bridged aza-[*n*.2.1]-indole-based tropanes in most examples, but was also extended to indole-fused medium-sized heterocycles **68** (Scheme 25). Chirality transfer from the substrates to the desired products allowed excellent enantioselectivitiy and diastereoselectivity.

### 3.2. Carbon-Heteroatom Bond Formation

#### 3.2.1. C–N Bond Formation

##### N-arylation

**Scheme 26:**
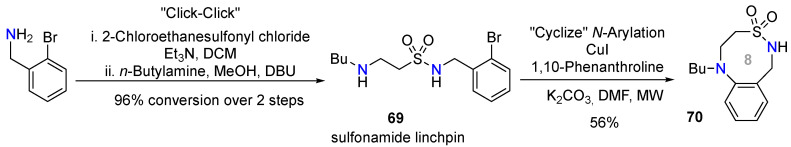
Synthesis of a sultam skeleton via Cu-catalyzed intramolecular *N*-Arylation.

Using a reagent-based diversity-oriented-synthesis strategy named “click, click, cyclize”, Hanson’s team built a library of sultam (cyclic sulfonamide) compounds [67]. They were able to produce a variety of 5- to 8-membered *S*-, *N,S*-, and *N*-heterocycles by exploring cyclization via alkylation, carbonylation, ring-closing metathesis, and copper-catalyzed *N*-arylation. A sulfonamide linchpin **69** substrate was prepared in two steps from 2-bromobenzylamine, then submitted to an intramolecular *N-*arylation by treatment with 1,10-phenanthroline and CuI under microwave irradiation to afford the 8-membered sultam **70** at 56% yield (Scheme 26).

**Scheme 27:**
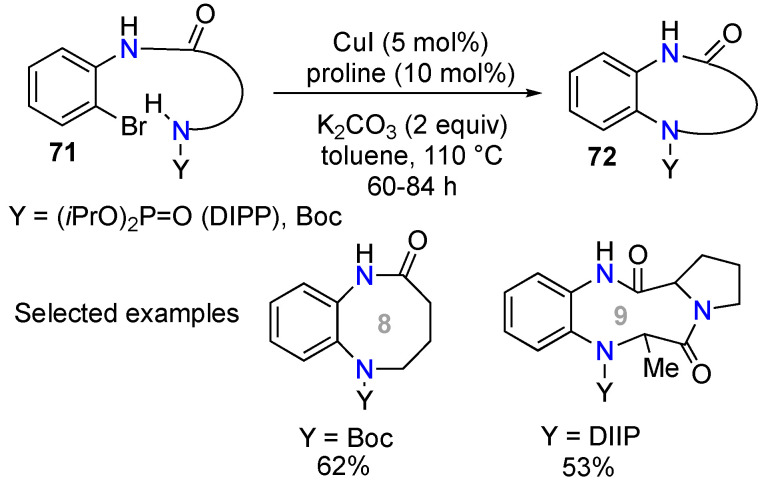
Synthesis of medium *N*-heterocycles via Cu-catalyzed intramolecular *N*-arylation.

Zhao and co-workers designed phosphoramidate and carbamate **71** derivatives that were efficiently transformed into medium (8 to 10 atoms) and large-sized *N*-heterocycles (12 and 16 atoms) upon submission to a copper-catalyzed intramolecular *N*-arylation [68]. The cyclization reaction conditions involved copper iodide and proline in toluene, giving access to *N*-heterocycle **72** (Scheme 27). The products were deprotected under acidic conditions. Intramolecular cyclization did not take place from the free amino group at the *N*-termini. Based on this result, the authors suggested that the presence of *N*-phosphoryl and *N*-Boc restrains substrate conformation and favors intramolecular *N*-arylation.

**Scheme 28:**
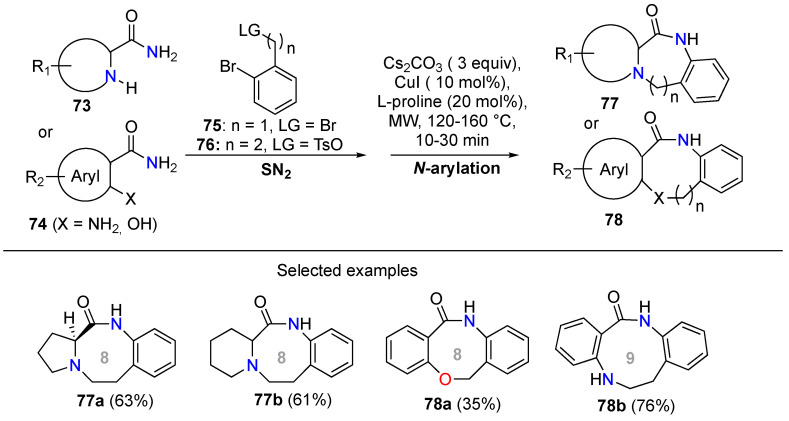
One-pot synthesis of medium-sized *N*-heterocycles via S_N_2 reaction followed by Cu-catalyzed intramolecular *N*-arylation.

Al-Tel and co-workers developed a one-pot procedure comprising two sequential steps, S_N_2 and *N*-arylation reactions, to access benzene-fused 8- and 9-membered *N*-heterocycles, most of which were tricyclic systems [69]. Structural diversity was provided by the selective S_N_2 step between a variety of **73** or **74** dinucleophiles and bromobenzene derivatives **75** or **76**. Among the selected ambident nucleophiles were pyrrolidine- and piperidine-2-carboxamide, 2-aminobenzamides, 2-aminothiophenol, and (1*H*-indol-2-yl)methanamine. The resulting substrates were directly involved in copper iodide- or L-proline-catalyzed cyclization via *N*-arylation under microwave irradiation, affording benzo-embedded medium-sized *N*-heterocycles **77** and **78** in moderate to good yields (Scheme 28).

##### N-Vinylation

**Scheme 29:**
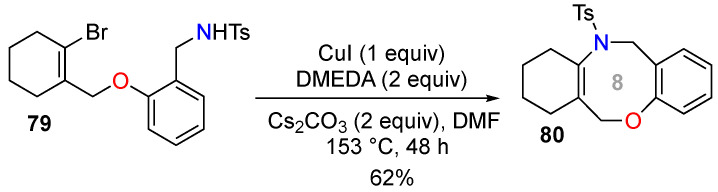
Synthesis of a benzoxazocine by Cu-catalyzed *N*-vinylation.

Li and co-workers reported a copper-catalyzed intramolecular C–N bond formation between alkenyl halides and sulfonamides providing 5-, 6-, 7-, and 8-membered heterocyclic enamines [70]. The best catalytic system consisted of CuI/N,N’-dimethylethylene-diamine (DMEDA), with Cs_2_CO_3_ as the base and DMF as the solvent. The endocyclic alkenyl halide **79** generated benzoxazocine **80** as an endocyclic medium-sized enamide (Scheme 29).

##### Hydroamination

**Scheme 30:**
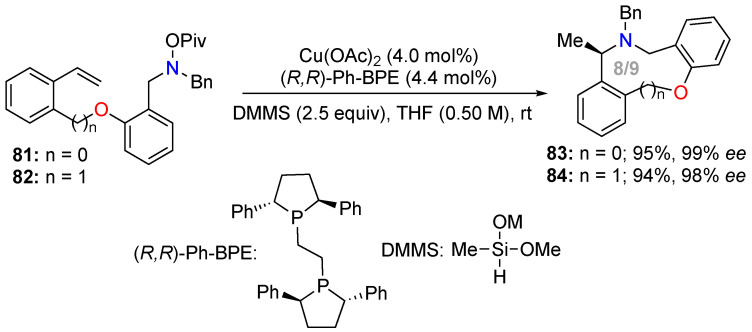
Asymmetric synthesis of medium-sized *N*,*O*-heterocycles via copper-catalyzed intramolecular hydroamination.

Buchwald and co-workers dedicated efforts to developing many strategies for copper-catalyzed hydroamination of olefins. An asymmetric intramolecular variant was proposed and applied to the synthesis of medium-sized *N*,*O*-heterocycles [71]. In this process, the chiral alkylcuprate formed by hydrocupration of the double bond reacted with an electrophilic nitrogen. The catalytic chiral copper species (CuH/L*) was formed from Cu(OAc)_2_ and dimethoxy(methyl)silane (DMMS) in the presence of an enantiopure bidentate phosphine ligand. The *N*-benzyl-*O*-pivaloylhydroxylamine moiety was used as the electrophilic nitrogen function, as this improved the yields and enantioselectivities of the reactions. This protocol was used for the enantioselective intramolecular hydroamination of the styryl double bond of substrates **81** and **82** to access benzoxazocine **83** and benzoxazonine **84**, respectively, with high yields and excellent enantiomeric excess (Scheme 30).

#### 3.2.2. C–O Bond Formation

**Scheme 31:**
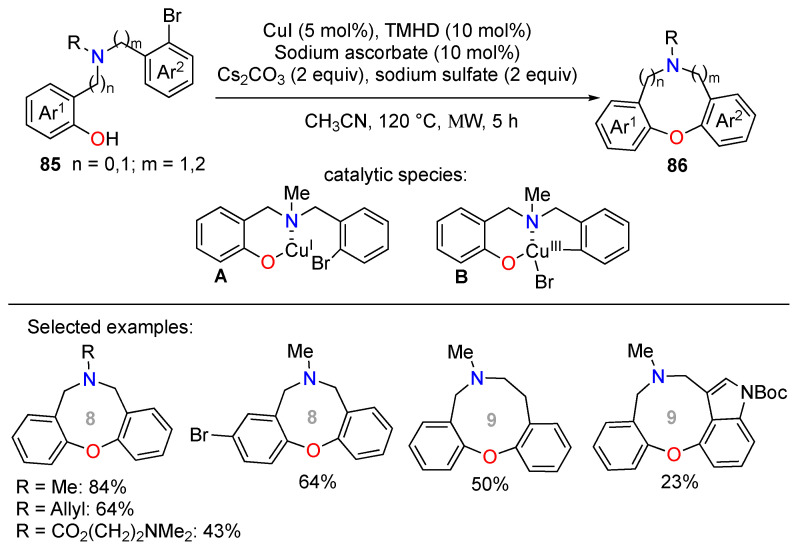
Copper-catalyzed intramolecular *O*-arylation to access dibenzoxazocines and oxazonines.

Based on previous work dealing with the synthesis of 7-, 8-, and 9-membered *N*-linked biaryl ring systems, Spring and co-workers [72] developed an *O*-linked version consisting of a copper-catalyzed intramolecular *O*-arylation to access biarylether-based oxazocines and oxazonines [73]. The acyclic **85** substrates are constituted from two aryl groups bearing an OH and a Br as an *ortho*-substituent, respectively, and tethered by an *N*-alkyl function (Scheme 31). Under the optimized reaction conditions involving the CuI/2,6-tetramethylheptanedione (TMHD) catalytic system, the *O*-arylation showed a broad substrate scope, accessing various biaryl-fused 8- and 9-membered *N*,*O*-heterocycle **86** products. Good to excellent yields were obtained for oxazocines excepting for those bearing ortho-substituents and for oxazonines. Complementary experiments demonstrated the crucial role of the substituted nitrogen atom present in the substrate structure. Indeed, the copper chelation by the nitrogen atom leads to a pre-organized species of type **A** that facilitates cyclization via the catalytic species **B**. A more flexible tertiary amine ligand placed as the *N*-substituent (R = CO_2_CH_2_CH_2_NMe_2_) was also efficient.

## 4. Methods Using Gold- or Silver-Catalyzed Reactions

Gold (I or III) complexes are the most effective catalysts for electrophilic activation of alkynes, with a wide field of synthetic applications; few other less-acidic late transition metals, such as Pt (II) or Ag(I), can be used as an alternative. The large tolerance of gold catalysis toward heteroatoms make gold catalysis a powerful and useful tool for the synthesis of functionalized molecular scaffolds, including heterocyclic cores [74].

### 4.1. Carbon–Carbon Bond Formation

**Scheme 32:**
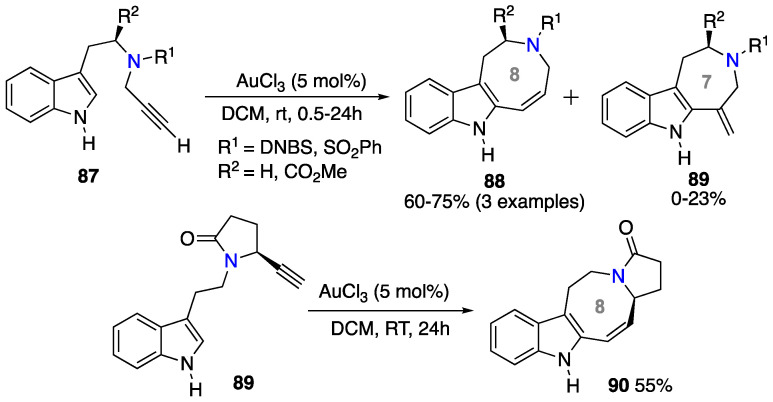
Gold-catalyzed cyclization of alkynylindole.

Echavarren and co-workers described gold-catalyzed hydroarylation of alkynylindole **87** (Scheme 32) [75,76]. As is often the case, a competition between *exo*- and *endo*-cyclization was observed. The regioselectivity was controlled by the oxidation state of the gold catalyst. Indeed, indoloazocine **88** products were obtained as major products with the gold(III) catalyst, while indoloazepine **89** products were obtained mostly with the gold(I) catalyst. The same authors applied the methodology for the preparation of **90**, a 1*H*-azocino[5, 4-*b*]indole skeleton of lundurines A-D [77].

**Scheme 33:**
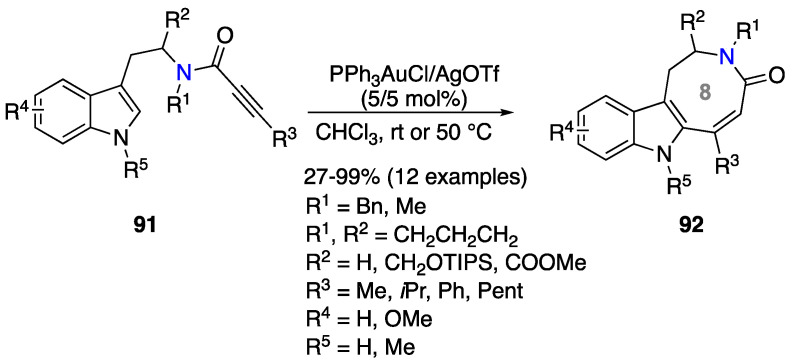
Gold-catalyzed alkyne hydroarylation of propargyl amide derivatives.

On their side, Eycken and co-workers obtained indoloazocine derivatives **92** via a cationic gold(I)-catalyzed alkyne hydroarylation of propargyl amide derivatives **91** (Scheme 33) [78]. A substrate scope expansion was achieved via the application of the method on some previously unreactive substrates and substrates bearing additional substituents on the indole core.

**Scheme 34:**
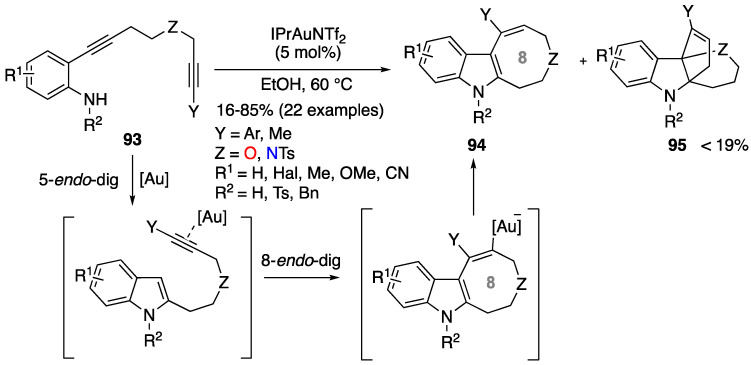
Gold-catalyzed cascade reactions of aniline derivatives.

Ohno and co-workers reported gold(I)-catalyzed cascade reactions of anilines **93** to form eight-membered ring-fused indoles **94** or propellane-type indoline 95 (Scheme 34) [79]. Depending on the reaction conditions, with IPr ligands and protic solvents such as ethanol, the formation of eight-membered ring-fused indoles **94** is favored. The reaction proceeded through an activation of alkyne by cationic gold, which promoted a 5-*endo*-dig hydroamination followed by 8-*endo*-dig hydroarylation.

**Scheme 35:**
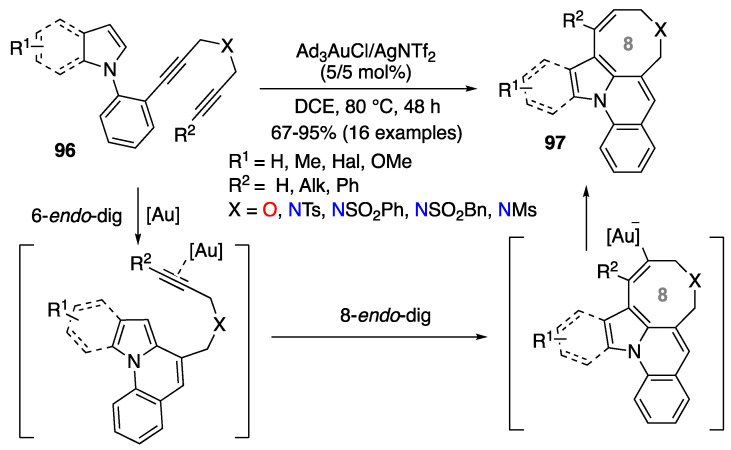
Gold-catalyzed cascade reactions of indole derivatives.

Shi and co-workers developed gold-catalyzed cascade reactions for the construction of eight-membered ring-fused indolizines **97** from indoles or pyrroles **96** (Scheme 35) [80]. The cascade reaction proceeded through two-fold hydroarylations (6-*endo*-dig and 8-*endo*-dig) in the presence of the gold(I)- catalyst.

**Scheme 36:**
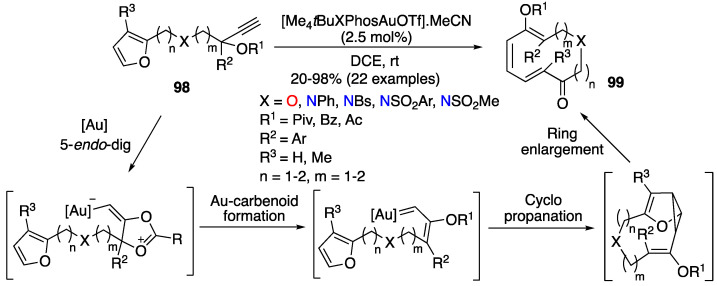
Gold-catalyzed cascade reactions for the construction of ten- and eleven-membered rings.

The challenging synthesis of medium-sized heterocycles possessing a *trans* double bond was described by Tang, Shi, and co-workers (Scheme 36) [81]. They described a gold(I)-catalyzed 1,2-acyloxy migration–intramolecular cyclopropanation–ring enlargement cascade reaction. The reaction provided access to ten- and eleven-membered *O*- or *N*-heterocycle **99** products and was highly chemo-selective at the C5 position of furan **98**.

**Scheme 37:**
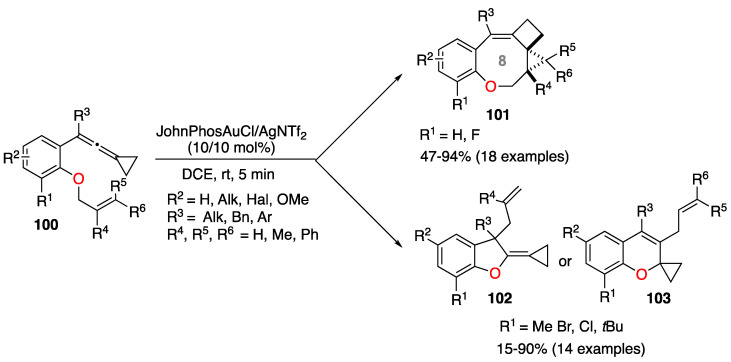
Gold(I)-catalyzed cycloisomerization of vinylidenecyclopropane-enes.

The same authors described a gold(I)-catalyzed cycloisomerization of vinylidenecyclopropane-ene **100** for the construction of *O*-heterocycles **101**–**103** through controllable carbene or non-carbene processes (Scheme 37) [82]. Depending on the substituents adjacent to the oxygen atom (R^1^ = H, F), a gold carbene is generated via rearrangement of vinylidenecyclopropane, giving access to eight-membered ring compounds **101**. The non-carbene processes allow the formation of five- or six-membered rings **102** and **103** through allyl migration.

**Scheme 38:**
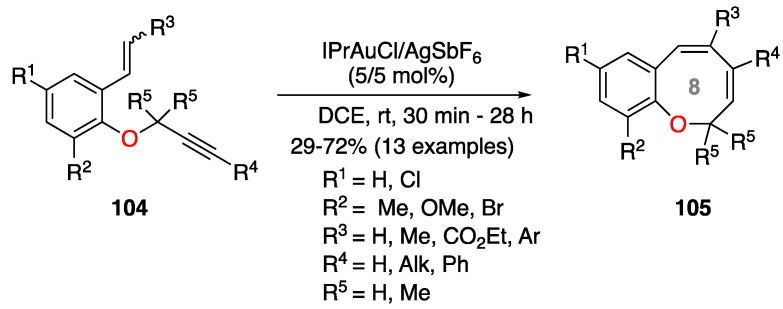
Synthesis of benzoxocines by gold(I)-catalyzed 8-*endo*-dig cyclization.

Kumar, Waldmann, and co-workers developed a rare 8-*endo*-dig cyclization reaction of *O*-propargyloxy styrene **104** products catalyzed by gold(I) complexes (Scheme 38) [83]. The transformation provides access to eight-membered ring **105**.

**Scheme 39:**
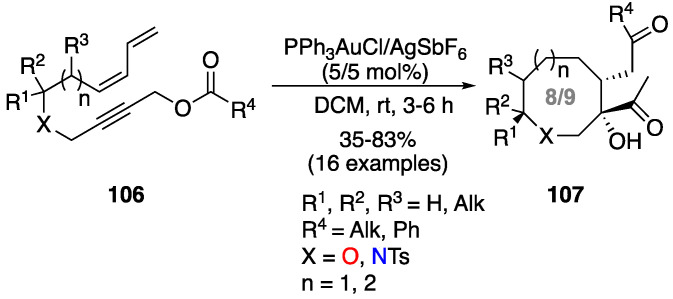
Synthesis of 8- and 9-membered ring ethers and amines by gold(I)-catalyzed cascade reaction.

She and co-workers reported the formation of eight- or nine-membered ring ethers and amines of **107** (Scheme 39) [84]. The strategy is based on a gold(I)-catalyzed cascade reaction involving enynyl ester **106** isomerization and intramolecular [3+2] cyclization. The geometry of the olefinic bond has an influence on this transformation, as only *Z* olefins underwent this reaction, while *E* olefins resulted in decomposition of the starting material.

### 4.2. Carbon–Heteroatom Bond Formation

#### C–O Bond Formation

The hydroalkoxylation of alkynes is the most convenient method for the synthesis of *O*-heterocycles. The high alkynophilicity of gold complexes allows these reactions, providing either the *exo*-dig or *endo*-dig product. However, few examples of 8-membered *O*-heterocycles are described and they are often in competition with 7-membered *O*-heterocycles.

**Scheme 40:**
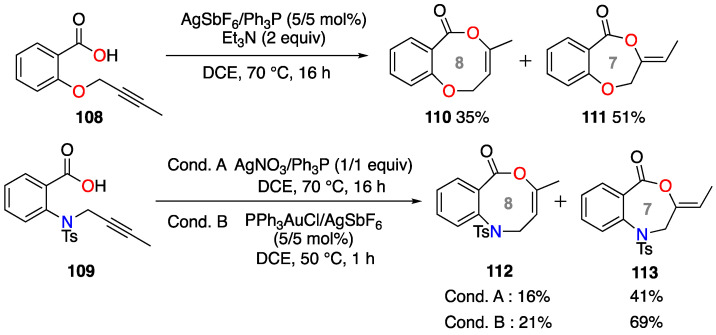
Ag(I)- and Au(I)-catalyzed hydroalkoxylation of alkynoic acids.

During their study on the synthesis of alkylidene lactone with silver(I) or gold(I) catalysts, Porcel and co-workers observed the formation 8-membered *O*-heterocycle **110** and *N*,*O*-heterocycle **112** (Scheme 40) [85]. Indeed, while terminal alkynoic acids were regioselectively transformed into 7-membered *O*- or *N*,*O*-heterocycles, the hydroalkoxylation of non-terminal alkynoic acids **108** and **109** was not regioselective, and the 8-membered *O*- or *N*,*O*-heterocycles were observed as minor products.

**Scheme 41:**
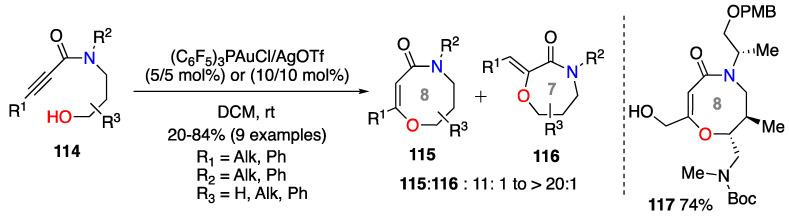
Au(I)-catalyzed hydroalkoxylation for oxazocenone synthesis.

Schreiber and co-workers described the development of a gold(I)-catalyzed 8-*endo*-dig hydroalkoxylation of alkynamide **114** products with good regioselectivities in favor of the 8-membered ring (up to 20:1) in order to form oxazocenone **115** (Scheme 41) [86]. This method was applied to a substrate with higher structural complexity to obtain **117**, an analog of a previously described bioactive benzoxazocenone [87,88].

**Scheme 42:**
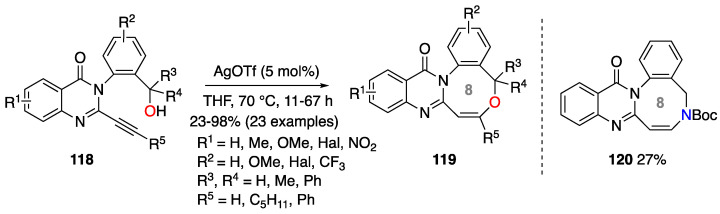
Ag(I)-catalyzed 8-*endo*-dig cyclization for quinazolinone-fused 8-membered heterocycle synthesis.

A recent publication referred to silver-catalyzed hydroalkoxylation of C2-alkynyl quinazolinone 118 for the synthesis of quinazolinone-fused, eight-membered *N*,*O*-heterocycle **119** (Scheme 42) [89]. The authors made mechanistic studies revealing that the silver catalyst might be involved in bidentate coordination of the imine group and alkyne to favor 8-*endo*-dig cyclization. It is interesting to note that they also synthesized one benzodiazocine **120** product at 27% yield.

**Scheme 43:**
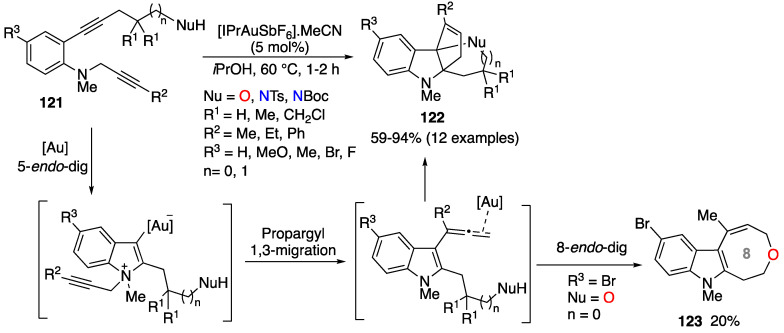
Au(I)-catalyzed cyclization of 2-alkynyl-*N*-propargylanilines.

On their side, Ohno and co-workers developed a gold(I)-catalyzed cascade reaction of 2-alkynyl-*N*-propargylaniline **121** by rearrangement of the propargyl group, providing access to fused indoline **122** (Scheme 43) [90]. During their study, the relatively low nucleophilicity of the indole bearing a bromo-substituent induced a side reaction and the production of the 8-membered *O*-heterocycle **123** in 20% yield.

## 5. Conclusions

This review has shown that the synthesis of medium-sized heterocycles by transition-metal-catalyzed intramolecular carbon–carbon or carbon–heteroatom bond formation represents an active area of research. Due to the interest aroused by these compounds in the field of drug discovery, other innovative methods will no doubt emerge to access these structures.

## Figures and Tables

**Table 1:** Selected examples of medium-sized heterocycles obtained by intramolecular Heck reaction.

Entry	Product ^a^	Reaction Conditions	Yields (%) (Examples) ^b^	Ref.
1	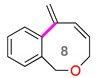	cat. PdCl_2_(PPh_3_)_2_, K_2_CO_3_, EtOH, DMF, 100 °C, 2 h	52	[19]
2	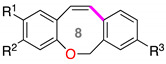	cat. Pd(OAc)_2_, AcOK, TBAB, DMF, 100 °C, 4 h	60–86 (10)	[20]
3	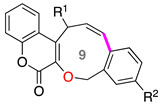	cat. Pd(OAc)_2_, K_2_CO_3_, MeCN, 80 °C, 1 h	61–70 (4)	[21]
4	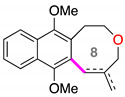	cat. Pd(OAc)_2_/PPh_3_, Cs_2_CO_3_, TBAC, DMF, 90 °C, 2 h	80–84%	[22]
5	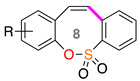	cat. Pd(OAc)_2_, AcOK, TBAB, DMF, 100 °C, 4 h	79–91 (4)	[23]
6	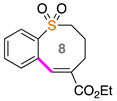	cat. Pd(OAc)_2_, TEAC, Cy_2_NMe, DMA, 100 °C, 12 h	69	[24]
7	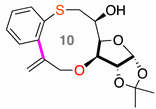	cat. Pd(OAc)_2_, Cs_2_CO_3_, TBAB, DMF, 100 °C, 12 h	59	[25]
8	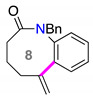	cat. Pd_2_(dba)_3_.CHCl_3_, PPh_3_, NEt_3_, DMF, 130 °C	72	[26]
9	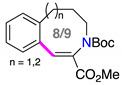	cat. Pd(OAc)_2_, NaHCO_3_, TBAC, DMF, 110 °C, 16 h	73–86 (2)	[27]
10	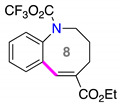	cat. Pd(OAc)_2_, TEAC, Cy_2_NMe, DMA, 95 °C, 6 h	72	[24]
11	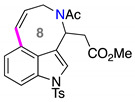	cat. Pd(OAc)_2_, PPh_3_, NEt_3_, MeCN, MW (125 °C), 6 h	85	[28]
12	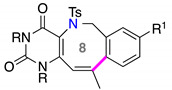	cat. Pd(OAc)_2_, AcOK, TBAB, DMF	80–85 (5)	[29]
13	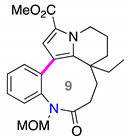	cat. Pd(OAc)_2_/DavePhos, K_2_CO_3_, MeCN, 80 °C, 1 h	47	[30]
14	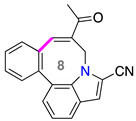	cat. Pd(OAc)_2_/JohnPhos, TBAA, DMF, 100 °C	60	[31]

^a^ The formed bond is indicated in magenta. ^b^ Number of examples.
